# Comprehensive geriatric assessment identifies multidimensional vulnerabilities in older adults with heart failure

**DOI:** 10.55730/1300-0144.6202

**Published:** 2026-01-29

**Authors:** Cihan KILIÇ, Rengin DEMİR, Özlem YILMAZ AYKENT, Zerrin YİĞİT, Pınar KÜÇÜKDAĞLI, Banu ÖZULU TÜRKMEN, Gülistan BAHAT, Mehmet Akif KARAN

**Affiliations:** 1 Division of Geriatrics, Department of Internal Medicine, İstanbul Faculty of Medicine, İstanbul University, İstanbul, Turkiye; 2 Department of Cardiology, Cardiology Institute, İstanbul University- Cerrahpaşa, İstanbul, Turkiye; 3 Division of Geriatrics, Department of Internal Medicine, İstanbul Education Research Hospital, İstanbul, Turkiye; 4 Division of Geriatrics, Department of Internal Medicine, Şişli Hamidiye Etfal Education and Research Hospital, İstanbul, Turkiye

**Keywords:** Heart failure, older adults, comprehensive geriatric assessment, frailty, functional capacity, geriatric syndromes

## Abstract

**Background/aim:**

Heart failure (HF) in older adults is frequently accompanied by geriatric syndromes that remain unrecognized in conventional cardiac evaluations. The vulnerabilities of older adults with and without HF in terms of muscle strength, physical performance, functional capacity, frailty, nutritional status, cognition, mood, anxiety, polypharmacy, and comorbidity were investigated using comprehensive geriatric assessment (CGA).

**Materials and methods:**

A cross-sectional study was conducted in two academic outpatient clinics, enrolling 420 adults aged ≥60 years, including 92 (21.9%) with HF. Data included sociodemographic characteristics, anthropometric measurements, and CGA parameters: handgrip strength, the Five-Times Sit-to-Stand Test (FTSST), the Short Physical Performance Battery (SPPB), activities of daily living (ADL), instrumental ADL (IADL), the FRAIL scale, the Mini Nutritional Assessment–Short Form, the Mini-Cog, the Geriatric Depression Scale–Short Form, and the Generalized Anxiety Disorder-7. Univariate and multivariate regression analyses were performed.

**Results:**

HF participants had slower FTSST (12.7 s versus 11.7 s; p = 0.002), and lower SPPB scores (10.0 versus 11.0; p = 0.016). IADL scores were significantly lower in the HF group (median, 7.0 versus 8.0; p < 0.001), indicating a higher prevalence of IADL impairment. Frailty (31.5% versus 18.9%; p = 0.009), malnutrition (6.5% versus 2.1%; p = 0.043), cognitive impairment (29.3% versus 14.3%; p = 0.001), and polypharmacy (88.0% versus 57.9%; p < 0.001) were significantly more prevalent in the HF group. Low lower extremity muscle strength (OR = 3.713), impaired IADL (OR = 4.974), and higher comorbidity burden (OR = 1.603) were identified as independent factors associated with HF in multivariate analysis.

**Conclusion:**

Older adults with HF showed substantial deficits across multiple CGA domains, including functional capacity, frailty, nutrition, and cognition. Integration of CGA into HF management may facilitate the identification of previously unrecognized vulnerabilities and support multidisciplinary care planning.

## 1. Introduction

Heart failure (HF) is a prevalent and complex clinical syndrome that disproportionately affects older adults, with prevalence increasing steeply with age. In high-income countries, HF affects approximately 1%–2% of the adult population and rises to over 10% among individuals aged 70 years or older [1]. Advances in acute cardiovascular care have improved survival from myocardial infarction and valvular disease, resulting in a growing population of older HF patients with multiple comorbidities and geriatric syndromes [2]. In this population, HF is rarely observed as an isolated condition. It frequently coexists with frailty, loss of skeletal muscle mass and function (so-called sarcopenia), cognitive impairment, and polypharmacy, all of which adversely influence prognosis, functional independence, and quality of life [3]. Importantly, several interventions are available to mitigate these vulnerabilities when they are recognized in a timely manner. The management of HF in older adults is further complicated by multimorbidity, making individualized assessment essential [4].

The Comprehensive Geriatric Assessment (CGA) is a multidimensional, interdisciplinary diagnostic process designed to evaluate medical, psychosocial, and functional capabilities of older adults. In cardiovascular populations, CGA can identify vulnerabilities such as malnutrition, frailty, cognitive decline, impaired mobility, and limited physical performance, which may not be detected in conventional cardiology evaluations [5]. Frailty and functional impairment are particularly relevant in HF because they predict hospital readmissions, reduced treatment tolerance, and higher mortality [6]. Physical performance metrics, such as gait speed and grip strength, have prognostic utility comparable to conventional exercise tests, highlighting the importance of functional evaluation in routine care.

Despite the clear overlap between HF and geriatric syndromes, few studies have directly compared CGA components between older adults with and without HF in real-world outpatient settings. Understanding these differences is crucial for tailoring multidisciplinary care models aimed at optimizing function, minimizing disability, and improving quality of life.

The relationship between HF and CGA domains—including muscle strength, physical performance, functional capacity, and geriatric syndromes—was investigated in older adults. It was hypothesized that older adults with HF would exhibit lower muscle strength, reduced functional capacity, and a higher prevalence of geriatric syndromes compared with those without HF, thereby emphasizing the potential for improved outcomes through timely identification and management.

## 2. Materials and methods

This was a cross-sectional observational study conducted in accordance with the Strengthening the Reporting of Observational Studies in Epidemiology (STROBE) guidelines. The study was conducted at two academic medical centers in İstanbul, Türkiye: the İstanbul Medical Faculty, Division of Geriatrics, and the İstanbul University-Cerrahpaşa Cardiology Institute. Both centers operate specialized outpatient clinics, which are staffed by geriatricians at the former institution and cardiologists at the latter.

### 2.1. Participants

The study was conducted between June 2017 and March 2020. Figure presents the study flow diagram. During the study period, 5920 patients visited outpatient clinics. The cardiology and geriatrics outpatient clinics had five examination rooms; however, only one room was available for Comprehensive Geriatric Assessment (CGA). Furthermore, each CGA required approximately twice as much time as a standard patient examination. Therefore, CGA was performed in 612 of these patients.

The inclusion criteria were as follows: age ≥60 years, ability to complete all CGA assessments, and provision of informed consent. The exclusion criteria were as follows: severe musculoskeletal, neurological, or psychiatric disorders that could impair physical performance assessment (e.g., advanced dementia, major depression, or psychosis) and acute trauma or conditions affecting handgrip strength measurement (e.g., hand tendon injury, joint edema, or recent fracture). After applying exclusion criteria, 192 individuals were excluded, leaving a final analytic sample of 420 participants, of whom 92 (21.9%) had a clinical diagnosis of HF.

### 2.2. Data collection and procedures

Sociodemographic data (age, sex, marital status, education, smoking, alcohol use), anthropometric measurements (height, weight), and clinical information (comorbidities, medication use) were recorded via structured interviews and medical record review. All CGA evaluations were performed by a trained and experienced healthcare professional (a physiotherapist) in a single session lasting approximately 60 min.

### 2.3. Heart failure diagnosis

The diagnosis of HF was confirmed based on clinical evaluation, echocardiographic parameters, and the New York Heart Association (NYHA) functional classification, as documented by cardiologists. Left ventricular ejection fraction (LVEF) was recorded.

### 2.4. Comprehensive geriatric assessment measures

#### 2.4.1. Muscle strength

Upper extremity muscle strength was measured by handgrip strength (HGS) using a Jamar hydraulic dynamometer (Performance Health, Warrenville, IL, USA) in a standardized seated position. The highest value from three attempts per hand was recorded. Population- and sex-specific cut-offs for low muscle strength were defined as <35 kg for men and <20 kg for women [7].

Lower extremity muscle strength was assessed using the Five-Times Sit-to-Stand Test (FTSST), with a performance time >15 s indicating low lower extremity strength [8].

#### 2.4.2. Physical performance

Physical performance was evaluated using the Short Physical Performance Battery (SPPB), which includes assessments of gait speed, balance, and chair rise time. Scores ≤8 were classified as poor performance [9].

#### 2.4.3. Functional capacity

Activities of daily living (ADL) were assessed using the Katz Index [10]. The ADL questionnaire assesses six basic activities of daily living: bathing, dressing, toileting, transferring, eating, and continence. Each ADL item is scored as 0 for dependence and 1 for independence, with a maximum total score of 6.

Instrumental activities of daily living (IADL) were measured with the Lawton–Brody scale [11]. The IADL questionnaire assesses eight instrumental activities of daily living: telephone use, shopping, meal preparation, housekeeping, laundry, transportation, medication management, and financial management. Each IADL item was scored as 0 for dependence and 1 for independence, with a maximum total score of 8. Scores <6 on the ADL or <8 on the IADL were considered indicative of functional impairment.

#### 2.4.4. Frailty

Frailty was evaluated using the FRAIL scale; scores ≥3 indicated frailty [12].

#### 2.4.5. Nutritional status

Nutritional status was evaluated using the Mini Nutritional Assessment–Short Form (MNA-SF); scores <8 indicated malnutrition [13].

#### 2.4.6. Cognitive status

Cognitive status was screened using the Mini-Cog test; abnormal recall or an abnormal clock-drawing result was considered suggestive of cognitive impairment [14].

#### 2.4.7. Mood and anxiety

Depressive symptoms were evaluated using the 15-item Geriatric Depression Scale–Short Form; scores ≥5 indicated depression [15]. Anxiety symptoms were screened using the Generalized Anxiety Disorder-7 (GAD-7); scores ≥10 indicated clinically relevant anxiety [16].

#### 2.4.8. Polypharmacy and comorbidity

Polypharmacy was defined as the use of ≥5 chronic medications [17]. Comorbidity burden was recorded as the total number of chronic diseases.

#### 2.4.9. Physical activity

Physical activity was classified according to the World Health Organization (WHO)^1^ recommendations; physical inactivity was defined as <150 min per week of moderate-intensity activity.

Participants were also asked whether they had experienced sleep problems, urinary incontinence, recent weight loss, or falls.

The study was approved by the Clinical Research Ethics Committee of İstanbul University, İstanbul Faculty of Medicine (approval no. 2017/550). All participants provided written informed consent. All study procedures were conducted in accordance with the Declaration of Helsinki.

#### 2.4.10. Statistical analysis

Data distribution was assessed using the Kolmogorov–Smirnov test. Continuous variables are presented as mean ± standard deviation (SD), and categorical variables are presented as numbers and frequencies (%). Group comparisons were performed using the Mann–Whitney U test for continuous variables and the chi-square test for categorical variables. Variables that were significant in the univariate analysis were entered into a multivariate logistic regression model to identify independent predictors of HF. Odds ratios (ORs) with 95% confidence intervals (CIs) were reported. Statistical analyses were performed using IBM SPSS Statistics (v21.0; IBM Corp., Armonk, NY, USA). A two-tailed p < 0.05 was considered statistically significant.

#### 2.4.11. Power analysis

Because no published data were available reporting muscle strength or functional performance outcomes (e.g., means or proportions) separately for older adults with and without HF, an a priori sample size calculation could not be conducted. Therefore, a post hoc power analysis was performed based on the collected data. To enhance statistical power, the non-HF control group was sampled at an approximate ratio of 4:1 relative to the HF group.

A post hoc power analysis was conducted for the independent-samples t-test comparing IADL scores between groups. The mean IADL score in the HF group was 6.0 (SD = 2.2; n = 92), while in the non-HF group it was 6.9 (SD = 2.0; n = 328). Based on these values, the calculated effect size (Cohen’s d) was 0.43. Using G*Power (version 3.1.9.7; Heinrich-Heine-Universität, Düsseldorf, Germany), the post hoc power (1–β) for a two-tailed independent-samples t-test was calculated as 95.0% at an alpha level of 0.05. These results indicate that the sample size was sufficient to detect a statistically significant difference between the groups.

## 3. Results

Table 1 presents the basic sociodemographic and clinical characteristics of the participants.

A total of 420 older adults (median age, 73 years; 61% women) were included in the study. Among the participants, 92 (21.9%) had heart failure (HF group), whereas 328 (78.1%) did not (non-HF group).

Participants in the HF group were more frequently male (72.8% versus 27.2%; p < 0 .001) and slightly younger than those in the non-HF group (median age, 70 versus 73 years; p < 0.001). No significant difference was observed in body mass index (BMI) between the groups (p = 0 .724). Marital status differed significantly between the groups, with a greater proportion of participants in the HF group being married (79.3% versus 58.2%; p < 0.001). Smoking (52.2% versus 31.1%; p < 0.001) and alcohol use (33.7% versus 12.2%; p < 0.001) were also more prevalent in the HF group (Table 1).

### 3.1. Cardiac function in the HF group

Table 2 presents the cardiac function characteristics of the HF group.

Among participants with HF, the mean LVEF was 36% (range, 15%–60%). LVEF was higher in women than in men (median, 40% versus 34%; p < 0.001). The median New York Heart Association (NYHA) functional class was II (range, I–III), with no significant sex-based differences (p = 0.197) (Table 2).

### 3.2. Comprehensive geriatric assessment outcomes

Table 3 presents the comprehensive geriatric assessment outcomes.

#### 3.2.1. Muscle strength

Upper extremity muscle strength measured by HGS was higher in the HF group (median, 32 kg versus 22 kg; p < 0.001). However, no significant sex-specific differences were observed when men and women were analyzed separately (men, p = 0.571; women, p = 0.131).

Lower extremity muscle strength assessed using the FTSST demonstrated longer completion times in the HF group in both men and women (men, median: 12.2 versus 11.1 s, p = 0.012; women, median: 14.9 versus 11.9 s, p < 0.001).

#### 3.2.2. Physical performance

Short Physical Performance Battery (SPPB) scores were lower in the HF group in both men and women (men, p = 0.029; women, p = 0.001).

#### 3.2.3. Functional capacity

Instrumental activities of daily living (IADL) scores were significantly lower in the HF group (p < 0.001), whereas basic activities of daily living (ADL) scores showed a smaller but statistically significant difference (p = 0.028).

#### 3.2.4. Frailty

Frailty prevalence was higher in the HF group (31.5% versus 18.9%; p = 0.009).

#### 3.2.5. Nutritional and cognitive status

Malnutrition prevalence, based on MNA-SF scores, was higher in the HF group (6.5% versus 2.1%; p = 0.043). Cognitive impairment, as identified by the Mini-Cog, was present in 29.3% of participants in the HF group compared with 14.3% in the non-HF group (p = 0.001).

#### 3.2.6. Mood and anxiety

Depressive symptoms were more common in the HF group but did not reach statistical significance (43% versus 38%; p = 0.534). GAD-7 scores were significantly higher in the HF group (median, 3 versus 2; p = 0.023).

#### 3.2.7. Polypharmacy and comorbid conditions

Higher rates of polypharmacy (≥5 medications: 88.0% versus 57.9%; p < 0.001) and a greater number of comorbidities (median, 4.5 versus 3.0; p < 0.001) were observed in the HF group. Sleep disturbances (48.9% versus 34.5%; p = 0.011) and recent weight loss (31.5% versus 13.7%; p < 0.001) were more prevalent in the HF group.

Coronary artery disease (67.4% versus 18.9%; p < 0.001), prior myocardial infarction (17.4% versus 0.6%; p < 0.001), arrhythmia (20.7% versus 11.6%; p = 0.025), diabetes mellitus (44.6% versus 32.6%; p = 0.034), chronic obstructive pulmonary disease (COPD) (15.2% versus 4.0%; p < 0.001), and chronic kidney disease (CKD) (14.1% versus 3.7%; p < 0.001) were more common in the HF group.

#### 3.2.8. Geriatric syndromes

The number of geriatric syndromes per patient was higher in the HF group (median, 5 versus 4; p = 0.040).

#### 3.2.9. Multivariate analysis

Table 4 presents the results of the multivariate logistic regression analysis.

In the multivariate logistic regression model, sex, age, number of comorbidities, lower extremity muscle strength, ADL status, IADL status, and weight loss were independently associated with HF as follows: male sex (OR = 0.107; 95% CI: 0.046–0.250; p < 0.001), age (OR = 0.896; 95% CI: 0.845–0-949; p < 0.001), higher number of comorbidities (OR = 1.578; 95% CI: 1.234–2.019; p < 0.001), low lower extremity muscle strength (OR = 3.124; 95% CI: 1.270–7.684; p = 0.013), impaired ADL (OR = 0.188; 95% CI: 0.071–0.495; p = 0.001), impaired IADL (OR = 4.700; 95% CI: 2.088–10.580; p < 0.001), and weight loss (OR = 2.547; 95% CI: 1.080–6.004; p = 0.033).

## 4. Discussion

The results of the present study indicate that HF in the older population was associated with reduced muscle strength, impaired physical performance, decreased functional independence, and a higher prevalence of geriatric syndromes, including malnutrition, cognitive impairment, polypharmacy, comorbidity, and frailty. These findings support the view that HF represents a multidimensional condition in older adults, requiring a holistic and interdisciplinary approach to management [2].

In the present study, older individuals with HF were more likely to be married, whereas those without HF were more likely to be divorced or widowed. Marriage may facilitate access to diagnosis and treatment through enhanced social support [18].

Furthermore, a higher prevalence of smoking and alcohol use was observed in the HF group. This finding is consistent with evidence indicating myocardial toxicity associated with alcohol consumption and the contribution of smoking to HF development through endothelial dysfunction [19,20]. These findings suggest that HF is associated not only with biological factors but also with social and behavioral determinants.

In the present study, women had significantly higher LVEF values than men. This finding aligns with existing evidence that heart failure with preserved ejection fraction (HFpEF) is more commonly observed in women. The predominance of HFpEF among women may be related to factors such as diastolic dysfunction, hypertension, obesity, and the cardioprotective effects of estrogen [1,2]. Therefore, higher LVEF values in women may not necessarily reflect a milder disease course but rather a different distribution of HF subtypes.

The proportion of men in the HF group was high (72.8%), and the median age of men with HF was lower (median, 70 versus 75 years). These findings may suggest that HF occurs at a younger age in men; however, disease severity cannot be inferred from these data. However, previous studies have reported conflicting results [21,22]. Therefore, the influence of sex on HF warrants further investigation.

In the present study, participants in the HF group were younger (median, 70.5 versus 73.0 years). The lower median age observed in the HF group may be related to the smaller sample size compared with the non-HF group and to the higher proportion of men in the HF group. It is possible that men with HF present to outpatient clinics at a younger age; however, symptom severity was not directly evaluated in this study. These findings underscore the importance of increased awareness of sex-related differences in HF. In a multicenter, descriptive national study, the proportion of elderly with HF aged 80 years and older was significantly higher [23]. As the global population ages and life expectancy among patients with HF increases due to advances in technology and treatment, the prevalence of HF among older adults has risen in recent years [1].

The prevalence of comorbidities, including ischemic heart disease, previous myocardial infarction, COPD, diabetes mellitus, and CKD, was significantly higher in the HF group. These findings suggest that HF is more frequently observed in individuals with a complex geriatric profile. In particular, the association between COPD and CKD and HF may be partially explained by shared hemodynamic alterations and systemic inflammation [24].

In the present study, older adults with HF demonstrated significantly lower handgrip strength, longer chair-stand times, and lower SPPB scores compared with those without HF. These findings are consistent with the literature identifying sarcopenia and physical deconditioning as common in HF, potentially related to reduced cardiac output, inflammation, hormonal alterations, and muscle atrophy [4]. Reduced muscle strength in HF is linked to poorer quality of life, higher readmission rates, and increased mortality [25], supporting its association with sarcopenia and impaired mobility. HF has also been associated with peripheral muscle atrophy [26].

Functionality independence represents a critical outcome for older adults. Functional independence, as assessed by ADL and IADL, was significantly impaired in the HF group. IADL impairment was particularly notable, with multivariate analysis demonstrating an approximately fivefold association with HF. This is consistent with findings by Rodríguez-Pascual et al., who reported that over 80% of older HF patients exhibit some degree of IADL limitation, strongly predicting hospitalization and mortality [27]. The relative preservation of ADL may be related to lower functional demands of basic activities (e.g., dressing and eating) compared with instrumental activities (e.g., shopping and transportation), rather than directly reflecting disease severity.

Cognitive impairment was present in nearly one-third of HF patients, almost twice that of the non-HF group. Cognitive deficits in HF are thought to be associated with cerebral hypoperfusion, microvascular injury, and systemic inflammation [28]. Importantly, cognitive impairment can impede medication adherence, self-care, and symptom recognition, further worsening outcomes. Given its high prevalence and potential adverse consequences, cognitive screening, although not mandatory for all older adults, should be strongly considered in older adults with HF.

Polypharmacy was significantly more prevalent in HF patients, reflecting both cardiovascular and noncardiovascular comorbidity burden. While appropriate pharmacotherapy is essential, polypharmacy increases the risk of drug–drug interactions, adverse effects, and hospitalizations. Optimizing medication regimens through CGA-guided medication review may mitigate these risks [3]. Therefore, recognition of polypharmacy indicates the need to assess whether medication use is appropriate or inappropriate. In this regard, explicit and implicit tools to assess medication appropriateness would be of use, such as STOPP/START criteria or TIME criteria [29,30].

Frailty was more prevalent among HF participants, consistent with metaanalyses reporting rates of 44%–50% [31]. This condition increases the risk of adverse outcomes by reducing physiological reserve, treatment tolerance, and resilience to stress. Its coexistence with HF is linked to nearly double the mortality risk [1].

Frailty requires individualized care approaches that may influence multiple aspects of HF management. Our results align with prior studies highlighting the high prevalence of frailty, sarcopenia, and functional impairment in older HF patients. Notably, Kamiya et al. found that gait speed in this population holds prognostic value comparable to the 6-min walk test, emphasizing the importance of functional assessment [6].

The present study expands on these findings by comparing a broad CGA profile between HF and non-HF older adults in a real-world clinical setting and by demonstrating the cumulative burden across multiple geriatric domains in HF.

Additionally, our results mirror the work by Ungar et al., who emphasized the predictive value of CGA in older cardiovascular patients undergoing procedures such as transcatheter aortic valve implantation [5]. Consistent with their findings, the integration of CGA into routine care may help anticipate risks and optimize outcomes in older HF populations. The relatively large sample size of the present study provided adequate statistical power. In addition, the assessment of geriatric syndromes such as depressive symptoms, anxiety, sleep disturbances, urinary incontinence, weight loss, and falls contributed to a more comprehensive evaluation.

These results of the present study emphasize the clinical relevance of nutritional deficits. Malnutrition prevalence, as assessed by the Mini Nutritional Assessment–Short Form (MNA-SF), was significantly higher in HF participants. Poor nutritional status in HF is multifactorial and may involve anorexia related to intestinal congestion, increased metabolic demand, and reduced nutrient absorption. Malnutrition is strongly linked to sarcopenia, functional decline, and increased mortality [32]. Optimal nutritional management has been shown to improve patient outcomes in various conditions, including HF, and is considered an essential component of clinical care [33].

These findings support the integration of multidisciplinary care models incorporating geriatric assessment into the routine management of HF in older adults. Such interventions may include exercise and rehabilitation programs targeting sarcopenia and reduced mobility [4], nutritional support to address malnutrition and cachexia [34], medication optimization to reduce polypharmacy-related risks [30], and cognitive and psychosocial support to enhance adherence and self-management.

Evidence from multidisciplinary cardiac rehabilitation trials suggests that such integrated approaches can improve physical function, reduce hospitalizations, and potentially improve survival in older HF patients [4]. However, underrecognition of these important issues may limit appropriate management, whereas timely identification may facilitate more effective intervention.

In the present study, recent unintentional weight loss emerged as a significant independent predictor of HF, nearly tripling the odds of an HF diagnosis (OR = 2.57). Among patients with HF, recent unintentional weight loss has been identified as a distinct prognostic marker associated with worse cardiac-related outcomes and reduced event-free survival [35]. These findings highlight the importance of monitoring nutritional status and addressing potential cachexia in older adults, particularly in the context of multimorbidity and functional decline.

### 4.1. Strengths and limitations

The present study has several limitations that should be acknowledged. First, the cross-sectional design precludes causal inference. HF may contribute to the development of geriatric syndromes, whereas geriatric syndromes may also influence the severity or clinical expression of HF, reflecting their bidirectional pathophysiological associations. Second, the study was conducted at two specialized centers, which may limit generalizability to community settings. Third, some variables such as physical activity were self-reported, potentially introducing recall bias. Finally, HF subtypes (HF with preserved, mildly reduced, and reduced ejection fraction) were not analyzed separately; future research is warranted to explore potential differences in geriatric profiles among these subgroups.

The study also has several strengths, including a relatively large sample size, the use of standardized CGA tools administered by a trained and experienced health care professional, and the inclusion of both HF and non-HF groups for comparison. The study was conducted in specialized geriatric and cardiology outpatient settings, allowing for high-quality assessments by experienced clinicians.

## 5. Conclusion

In conclusion, the present study demonstrated that older adults with HF exhibited significantly poorer performance across multiple domains of CGA, including muscle strength, functional status, and geriatric syndromes. These findings highlight the importance of integrating CGA into standard HF management to identify vulnerabilities, guide individualized interventions, and potentially optimize outcomes. A multidisciplinary, patient-centered approach appears essential for the comprehensive care of this growing and vulnerable population. Future research is warranted to include prospective studies evaluating the predictive value of CGA domains for hospitalization, functional decline, and mortality in HF, as well as additional interventional trials.

## Figures and Tables

**Figure:**
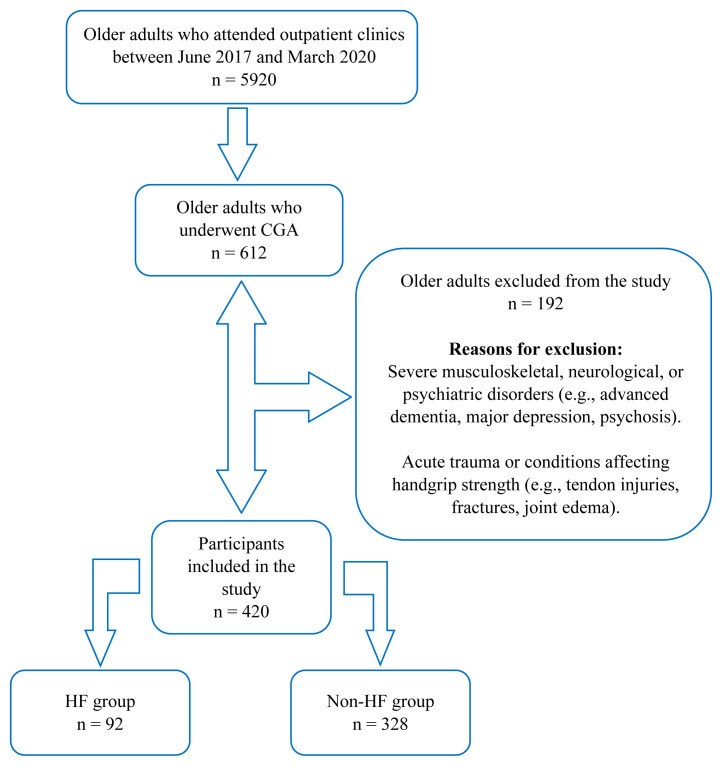
Study flow diagram.

**Table 1:** Baseline sociodemographic and clinical characteristics of the study participants.

Variable	Total (n = 420)	HF present (n = 92, 21.9%)	HF absent (n = 328, 78.1%)	p-value
**Sex** **^1^**	M: 164 (39%) F: 256 (61%)	M: 67 (72.8%) F: 25 (27.2%)	M: 97 (29.6%) F: 231(70.4%)	**<0.001**
**Age (years)** **^2^**	73.0 (60–88)	70.5 (60–88)	73.0 (60–88)	**<0.001**
	M: 73 (60–88)	M: 70 (60–88)	M: 75 (61–88)	**<0.001**
	F: 73 (60–88)	F: 71 (64–87)	F: 73 (60–88)	**0.552**
**BMI (kg/m** **^2^** **)** **^2^**	29.4 (18.0–58.8)	29.3 (18.0–47.0)	29.6 (18.3–58.8)	0.724
**Educational status**				
**Illiterate** **^1^**	108 (25.7%)	22 (23.9%)	86 (26.2%)	0.436
**Primary school** **^1^**	208 (49.5%)	52 (56.5%)	156 (47.6%)	
**High school** **^1^**	42 (10.0%)	8 (8.7%)	34 (10.4%)	
**Education beyond high school** **^1^**	62 (14.8%)	10 (10.9%)	52 (15.9%)	
**Marital status**				
**Single** **^1^**	6 (1.4%)	2 (2.2%)	4 (1.2%)	**<0.001**
**Married** **^1^**	264 (62.9%)	73 (79.3%)	191 (58.2%)	
**Divorced/widowed** **^1^**	150 (35.7%)	17 (18.5%)	133 (40.5%)	
**History of smoking ** **^1^**	150 (35.7%)	48 (52.2%)	102 (31.1%)	**<0.001**
**History of alcohol use ** **^1^**	71 (16.9%)	31 (33.7%)	40 (12.2%)	**<0.001**
**HGS** **^2^**	24.0 (6–50)	32.0 (8–50)	22.0 (6–50)	**<0.001**
**HGS** (Male)**^2^**	34.0 (6–50)	34.0 (18–50)	34.0 (6–50)	0.571
**HGS** (Female)**^2^**	20.0 (6–44)	20.0 (8–38)	22.0 (6–44)	0.132
**FTSST** **^2^**	11.9 (5.6–37.5)	12.7 (6.6–37.5)	11.7 (5.6–36.0)	**0.002**
**FTSST** (Male)**^2^**	11.5 (6.2–35.5)	12.2 (6.6–26.4)	11.1 (6.2–35.5)	**0.012**
**FTSST** (Female)**^2^**	12.4 (5.6–37.5)	14.9 (10.0–37.5)	11.9 (5.6–36.0)	**<0.001**
**SPPB** **^2^**	11.0 (1–12)	10.0 (1–12)	11.0 (1–12)	**0.016**
**SPPB** (Male)**^2^**	10.1 (1–12)	11.0 (1–12)	11.0 (4–12)	**0.029**
**SPPB** (Female)**^2^**	10.0 (2–12)	9.0 (3–12)	10.0 (2–12)	**0.001**
**ADL** **^2^**	6.0 (1–6)	6.0 (3–6)	6.0 (1–6)	**0.028**
**IADL** **^2^**	8.0 (0–8)	7.0 (0–8)	8.0 (0–8)	**<0.001**
**MNA-SF** **^2^**	13.0 (2–14)	13.0 (4–14)	13.0 (2–14)	0.215
**FRAIL** **^2^**	1.0 (0–5)	1.0 (0–4)	1.0 (0–5)	**0.013**
**GDS-SF** **^2^**	3.0 (0–14)	3.0 (0–14)	3.0 (0–14)	0.534
**GAD-7** **^2^**	2.0 (0–21)	3.0 (0–17)	2.0 (0–21)	**0.023**
**Number of comorbidities ** **^2^**	4.0 (0–9)	4.5 (0–9)	3.0 (2–9)	**<0.001**
**Number of medications** **^2^**	6.0 (0–21)	8.0 (1–16)	5.0 (0–21)	**<0.001**

p-value: statistical significance;^1^ n(%);^2^ median (minimum–maximum); HF: heart failure; M: male; F: female; BMI: body mass index (weight / height^2^). Illiterate: Did not complete primary school; primary education: primary and secondary school graduate; higher education: education beyond high school; HGS: handgrip strength (kg); FTSST: Five-Times Sit-to-Stand Test (s); SPPB: Short Physical Performance Battery (0–12 score); ADL: activities of daily living (0–6 score); IADL: instrumental activities of daily living (0–8 score); MNA-SF: Mini Nutritional Assessment–Short Form (0–14 score); FRAIL: FRAIL Scale (0–5 score); GDS-SF: Geriatric Depression Scale–Short Form (0–15 score); GAD-7: Generalized Anxiety Disorder-7 (0–21 score).

**Table 2:** Cardiac function in the HF group.

Variable	Total (n = 92)	Male (n = 67, 72.8%)	Female (n = 25, 27.2%)	p-value
**EF** **^2^**	36 (15–60)	34 (15–60)	40 (24–50)	**0.001**
**HFrEF** **^1^**	60 (65.2%)	48 (71.6%)	12 (48.0%)	**0.027**
**HFmrEF** **^1^**	25 (27.2%)	13 (19.4%)	12 (48.0%)	
**HFpEF** **^1^**	7 (7.6%)	6 (9.0%)	1 (4.0%)	
**NYHA** **^2^**	2 (1–3)	2 (1–3)	2 (2–3)	0.197

p-value: statistical significance;^1^ n(%);^2^ median (minimum–maximum); EF: ejection fraction; HFrEF: heart failure with reduced ejection fraction (<40% EF); HFmrEF: heart failure with mildly reduced ejection fraction (40%–49% EF); HFpEF: heart failure with preserved ejection fraction (≥50% EF); NYHA: New York Heart Association functional class.

**Table 3:** Comprehensive geriatric assessment outcomes.

Variable	Total (n = 420)	HF present (n = 92, 21.9%)	HF absent (n = 328, 78.1%)	p-value
**LUEMS** **^1^**	176 (41.9%)	49 (53.3%)	127 (38.7%)	**0.012**
**LLEMS** **^1^**	108 (25.7%)	31 (33.7%)	77 (23.5%)	**0.047**
**Low physical performance** **^1^**	107 (25.5%)	30 (32.6%)	77 (23.5%)	0.076
**Impaired ADL** **^1^**	106 (25.2%)	14 (15.2%)	92 (28%)	**0.012**
**Impaired IADL** **^1^**	166 (39.5%)	58 (63.0%)	108 (32.9%)	**<0.001**
**Physical inactivity** **^1^**	66 (15.7%)	19 (20.7%)	47 (14.3%)	0.141
**Malnutrition** **^1^**	13 (3.1%)	6 (6.5%)	7 (2.1%)	**0.043**
**Frailty** **^1^**	91 (21.7%)	29 (31.5%)	62 (18.9%)	**0.009**
**Cognitive impairment** **^1^**	74 (17.6%)	27 (29.3%)	47 (14.3%)	**0.001**
**Depressive symptoms** **^1^**	165 (39.3%)	40 (43.5%)	125 (38.1%)	0.351
**Anxiety** **^1^**	38 (9.0%)	11 (12.0%)	27 (8.2%)	0.633
**Polypharmacy** **^1^**	271 (64.5%)	81 (88.0%)	190 (57.9%)	**<0.001**
**Sleep disturbance** **^1^**	158 (37.6%)	45 (48.9%)	113 (34.5%)	**0.011**
**Urinary incontinence** **^1^**	176 (41.9%)	31 (33.7%)	145 (44.2%)	0.071
**Unintentional weight loss** **^1^**	74 (17.6%)	29 (31.5%)	45 (13.7%)	**<0.001** **^b^**
**Falls** **^1^**	161 (38.3%)	34 (37.0%)	127 (38.7%)	0.759
**Number of geriatric syndromes** **^2^**	4.0 (0–12)	5.0 (0–11)	4.0 (0–12)	**0.040**
**Comorbidities**				
**Ischemic heart disease** **^1^**	124 (29.5%)	62 (67.4%)	62 (18.9%)	**<0.001**
**Myocardial infarction** **^1^**	18 (4.3%)	16 (17.4%)	2 (0.6%)	**<0.001**
**Arrhythmia** **^1^**	57 (13.6%)	19 (20.7%)	38 (11.6%)	**0.025**
**Hypertension** **^1^**	296 (70.5%)	63 (68.5%)	233 (71.0%)	0.635
**Dyslipidemia** **^1^**	126 (30.0%)	35 (38.0%)	91 (27.7%)	0.057
**Cerebrovascular disease** **^1^**	22 (5.2%)	5 (5.4%)	17 (5.2%)	0.924
**Obesity** **^1^**	197 (46.9%)	41 (44.6%)	156 (47.6%)	0.611
**Diabetes mellitus** **^1^**	148 (35.2%)	41 (44.6%)	107 (32.6%)	**0.034**
**Dementia** **^1^**	37 (8.8%)	3 (3.3%)	34 (10.4%)	**0.034**
**COPD** **^1^**	27 (6.4%)	14 (15.2%)	13 (4.0%)	**<0.001**
**Chronic kidney disease** **^1^**	25 (6.0%)	13 (14.1%)	12 (3.7%)	**<0.001**

p-value: statistical significance;^1^ n(%);^2^ median (minimum–maximum); HF: heart failure; LUEMS: low upper extremity muscle strength (men <35 kg HGS; women <20 kg HGS); LLEMS: low lower extremity muscle strength (FTSST >15 s). Low physical performance: SPPB ≤8; Impaired ADL: activities of daily living (ADL) score <6; Impaired IADL: instrumental activities of daily living (IADL) score <8; Physical inactivity: <3 days and <150 min of physical activity per week; Malnutrition: MNA-SF score <8; Frailty: FRAIL score ≥3; Cognitive impairment: Mini-Cog test (+); Depressive symptoms: GDS-SF score ≥5; Anxiety: GAD-7 score ≥10; Polypharmacy: >5 medications; Unintentional weight loss: within the last 3 months; Falls: within the last year; Obesity: BMI ≥30 kg/m^2^; COPD: chronic obstructive pulmonary disease.

**Table 4:** Multivariate logistic regression analysis: Independent predictors of HF.

Independent variables	p value	Odds ratio	95% CI	
**Sex**	**<0.001**	**0.118**	**0.053**	**0.263**
**Age**	**<0.001**	**0.892**	**0.842**	**0.944**
History of smoking	0.992	0.996	0.465	2.134
History of alcohol use	0.075	2.318	0.918	5.852
**Number of comorbidities**	**<0.001**	**1.589**	**1.243**	**2.032**
Number of medications	0.196	1.095	0.954	1.256
**LLEMS**	**0.015**	**3.029**	**1.241**	**7.393**
**Impaired ADL**	**0.001**	**0.182**	**0.069**	**0.477**
**Impaired IADL**	**<0.001**	**4.500**	**2.014**	**10.055**
Malnutrition	0.892	1.134	0.185	6.949
Frailty	0.136	0.499	0.200	1.245
Cognitive impairment	0.489	1.336	0.587	3.040
Polypharmacy	0.195	2.000	0.701	5.706
Sleep disturbance	0.108	1.784	0.880	3.615
**Unintentional weight loss**	**0.030**	**2.574**	**1.097**	**6.038**

p-value: statistical significance; CI: confidence interval; Dependent variable: HF; HF: heart failure; LLEMS: low lower extremity muscle strength (FTSST >15 s). Impaired ADL: activities of daily living (ADL) score <6; Impaired IADL: instrumental activities of daily living (IADL) score <8; Malnutrition: MNA-SF score <8; Frailty: FRAIL score ≥3; Cognitive impairment: Mini-Cog test (+); Polypharmacy: number of medications ≥5; Unintentional weight loss: within the last 3 months.

## Data Availability

The datasets generated and/or analyzed during this study are not publicly available but are available from the corresponding author upon reasonable request.

## Footnotes

1 World Health Organization (2010). Global recommendations on physical activity for health: adults aged 65 years and above. [online] Website https://www.who.int/docs/default-source/physical-activity/information-sheet-global-recommendations-on-physical-activity-for-health/physical-activity-recommendations-65years.pdf (accessed 02.10.2024)

## References

[1] McDonagh TA Metra M Adamo M Gardner RS Baumbach A 2021 ESC Guidelines for the diagnosis and treatment of acute and chronic heart failure: Developed by the Task Force for the diagnosis and treatment of acute and chronic heart failure of the European Society of Cardiology (ESC) with the special contribution of the Heart Failure Association (HFA) of the ESC Revista Española de Cardiología (English Edition) 2022 75 6 523 10.1016/j.rec.2022.05.005 35636830

[2] Irabor B McMillan JM Fine NM Assessment and management of older patients with transthyretin amyloidosis cardiomyopathy: geriatric cardiology, frailty assessment and beyond Frontiers in Cardiovascular Medicine 2022 9 863179 10.3389/fcvm.2022.863179 35656395 PMC9152087

[3] Jepma P Verweij L Tijssen A Heymans MW Flierman I The performance of the Dutch Safety Management System frailty tool to predict the risk of readmission or mortality in older hospitalised cardiac patients BMC Geriatrics 2021 21 1 299 10.1186/s12877-021-02243-5 33964888 PMC8105911

[4] Kamiya K Sato Y Takahashi T Tsuchihashi-Makaya M Kotooka N Multidisciplinary cardiac rehabilitation and long-term prognosis in patients with heart failure Circulation: Heart Failure 2020 13 10 e006798 10.1161/CIRCHEARTFAILURE.119.006798 32986957

[5] Ungar A Mannarino G van der Velde N Baan J Thibodeau MP Comprehensive geriatric assessment in patients undergoing transcatheter aortic valve implantation – results from the CGA-TAVI multicentre registry BMC Cardiovascular Disorders 2018 18 1 1 10.1186/s12872-017-0740-x 29301486 PMC5755352

[6] Kamiya K Hamazaki N Matsue Y Mezzani A Corrà U Gait speed has comparable prognostic capability to six-minute walk distance in older patients with cardiovascular disease European Journal of Preventive Cardiology 2018 25 2 212 219 10.1177/2047487317735715 28990422

[7] Bahat G Kilic C Altinkaynak M Karan MA Comparison of standard versus population-specific handgrip strength cut-off points in the detection of probable sarcopenia after launch of EWGSOP2 Aging Male 2020 23 5 1564 1569 10.1080/13685538.2020.1870038 33432867

[8] Cesari M Kritchevsky SB Newman AB Simonsick EM Harris TB Added value of physical performance measures in predicting adverse health-related events: results from the Health, Aging and Body Composition Study Journal of the American Geriatrics Society 2009 57 2 251 259 10.1111/j.1532-5415.2008.02126.x 19207142 PMC2695653

[9] Pavasini R Guralnik J Brown JC di Bari M Cesari M Short Physical Performance Battery and all-cause mortality: systematic review and meta-analysis BMC Medicine 2016 14 1 215 10.1186/s12916-016-0763-7 28003033 PMC5178082

[10] Katz S Ford AB Moskowitz RW Jackson BA Jaffe MW Studies of illness in the aged. The Index of ADL: a standardized measure of biological and psychosocial function Journal of the American Medical Association 1963 185 914 919 10.1001/jama.1963.03060120024016 14044222

[11] Lawton MP Brody EM Assessment of older people: self-maintaining and instrumental activities of daily living The Gerontologist 1969 9 3 179 186 5349366

[12] Morley JE Malmstrom TK Miller DK A simple frailty questionnaire (FRAIL) predicts outcomes in middle aged African Americans The Journal of Nutrition, Health & Aging 2012 16 7 601 608 10.1007/s12603-012-0084-2 PMC4515112 22836700

[13] Rubenstein LZ Harker JO Salvà A Guigoz Y Vellas B Screening for undernutrition in geriatric practice: developing the short-form mini-nutritional assessment (MNA-SF) The Journals of Gerontology Series A: Biological Sciences and Medical Sciences 2001 56 6 M366 M372 10.1093/gerona/56.6.m366 11382797

[14] Borson S Scanlan J Brush M Vitaliano P Dokmak A The mini-cog: a cognitive ‘vital signs’ measure for dementia screening in multi-lingual elderly International Journal of Geriatric Psychiatry 2000 15 11 1021 1027 10.1002/1099-1166(200011)15:11<1021::aid-gps234>3.0.co;2-6 11113982

[15] Arthur A Jagger C Lindesay J Graham C Clarke M Using an annual over-75 health check to screen for depression: validation of the short Geriatric Depression Scale (GDS15) within general practice International Journal of Geriatric Psychiatry 1999 14 6 431 439 10.1002/(SICI)1099-1166(199906)14:6<431::AID-GPS937>3.0.CO;2-I 10398352

[16] Spitzer RL Kroenke K Williams JB Löwe B A brief measure for assessing generalized anxiety disorder: the GAD-7 Archives of Internal Medicine 2006 166 10 1092 1097 10.1001/archinte.166.10.1092 16717171

[17] Wong CY Chaudhry SI Desai MM Krumholz HM Trends in comorbidity, disability, and polypharmacy in heart failure The American Journal of Medicine 2011 124 2 136 143 10.1016/j.amjmed.2010.08.017 21295193 PMC3237399

[18] Manfredini R De Giorgi A Tiseo R Boari B Cappadona R Marital status, cardiovascular diseases, and cardiovascular risk factors: a review of the evidence Journal of Women’s Health 2017 26 6 624 632 10.1089/jwh.2016.6103 28128671

[19] Piano MR Alcohol’s effects on the cardiovascular system Alcohol Research 2017 38 2 219 241 28988575 10.35946/arcr.v38.2.06 PMC5513687

[20] Ambrose JA Barua RS The pathophysiology of cigarette smoking and cardiovascular disease: an update Journal of the American College of Cardiology 2004 43 10 1731 1737 10.1016/j.jacc.2003.12.047 15145091

[21] Sahle BW Owen AJ Mutowo MP Krum H Reid CM Prevalence of heart failure in Australia: a systematic review BMC Cardiovascular Disorders 2016 16 32 10.1186/s12872-016-0208-4 26852410 PMC4744379

[22] van Riet EE Hoes AW Wagenaar KP Limburg A Landman MA Epidemiology of heart failure: the prevalence of heart failure and ventricular dysfunction in older adults over time. A systematic review European Journal of Heart Failure 2016 18 3 242 252 10.1002/ejhf.483 26727047

[23] Gök G Zoghi M Sinan ÜY Kılıç S Tokgözoğlu L Demographics of patients with heart failure who were over 80 years old and were admitted to the cardiology clinics in Turkey Anatolian Journal of Cardiology 2019 21 4 196 205 30930455 10.14744/AnatolJCardiol.2018.94556 PMC6528498

[24] van Deursen VM Damman K van der Meer P Wijkstra PJ Luijckx GJ Co-morbidities in heart failure Heart Failure Reviews 2014 19 2 163 172 10.1007/s10741-012-9370-7 23266884

[25] Dai KZ Laber EB Chen H Mentz RJ Malhotra C Hand grip strength predicts mortality and quality of life in heart failure: insights from the Singapore cohort of patients with advanced heart failure Journal of Cardiac Failure 2023 29 6 911 918 10.1016/j.cardfail.2022.11.009 36526216

[26] Drescher C Konishi M Ebner N Springer J Loss of muscle mass: current developments in cachexia and sarcopenia focused on biomarkers and treatment Journal of Cachexia, Sarcopenia and Muscle 2015 6 4 303 311 10.1002/jcsm.12082 26676067 PMC4670737

[27] Rodríguez-Pascual C Paredes-Galán E Ferrero-Martínez AI Gonzalez-Guerrero JL Hornillos-Calvo M The frailty syndrome is associated with adverse health outcomes in very old patients with stable heart failure: a prospective study in six Spanish hospitals International Journal of Cardiology 2017 236 296 303 10.1016/j.ijcard.2017.02.016 28215465

[28] Yang M Sun D Wang Y Yan M Zheng J Cognitive impairment in heart failure: landscape, challenges, and future directions Frontiers in Cardiovascular Medicine 2022 8 831734 10.3389/fcvm.2021.831734 35198608 PMC8858826

[29] O’Mahony D Cherubini A Guiteras AR Denkinger M Beuscart JB STOPP/START criteria for potentially inappropriate prescribing in older people: version 3 European Geriatric Medicine 2023 14 4 625 632 10.1007/s41999-023-00777-y 37256475 PMC10447584

[30] Bahat G Ilhan B Erdogan T Halil M Savas S Turkish inappropriate medication use in the elderly (TIME) criteria to improve prescribing in older adults: TIME-to-STOP/TIME-to-START European Geriatric Medicine 2020 11 3 491 498 10.1007/s41999-020-00297-z 32297261 PMC7280176

[31] Denfeld QE Winters-Stone K Mudd JO Gelow JM Kurdi S The prevalence of frailty in heart failure: a systematic review and meta-analysis International Journal of Cardiology 2017 236 283 289 10.1016/j.ijcard.2017.01.153 28215466 PMC5392144

[32] de Pinho NB Martucci RB Rodrigues VD D’Almeida CA Thuler LCS Malnutrition associated with nutrition impact symptoms and localization of the disease: results of a multicentric research on oncological nutrition Clinical Nutrition 2019 38 3 1274 1279 10.1016/j.clnu.2018.05.010 29853223

[33] Yu X Chen Q Xu Lou I Dietary strategies and nutritional supplements in the management of heart failure: a systematic review Frontiers in Nutrition 2024 11 1428010 10.3389/fnut.2024.1428010 39464682 PMC11502353

[34] Bahat G Akmansu M Gungor L Halil M Bicakli DH Optimal use of oral nutritional supplements (ONS) in medical nutrition therapy: ONS consensus report from KEPAN European Journal of Clinical Nutrition 2023 77 7 705 709 10.1038/s41430-022-01229-9 36352101 PMC9645761

[35] Song EK Lee Y Moser DK Dekker RL Kang SM The link of unintentional weight loss to cardiac event-free survival in patients with heart failure Journal of Cardiovascular Nursing 2014 29 5 439 447 10.1097/JCN.0b013e3182a46ba8 24088622 PMC4130800

